# From Animals to Arthroplasty: Insights From Three Cases of *Pasteurella* Prosthetic Joint Infection and a Comprehensive Review of the Literature

**DOI:** 10.1155/cris/4246149

**Published:** 2026-02-18

**Authors:** Wankumbu Chisala, Matthew Saunders, Robert Townsend, David Partridge

**Affiliations:** ^1^ Department of Infection, Immunity and Cardiovascular Disease, The University of Sheffield, Sheffield, UK, sheffield.ac.uk; ^2^ The Florey Institute of Infection, The University of Sheffield, Sheffield, UK, sheffield.ac.uk

**Keywords:** DAIR (debridement antibiotics and implant retention), Pasteurella, prosthetic joint infection, zoonotic infection

## Abstract

*Pasteurella* spp. are rare but important zoonotic pathogens that can cause prosthetic joint infection (PJI). We present 3 cases of *Pasteurella* prosthetic joint infections (PJIs) following close animal contact that required treatment with DAIR (debridement, antibiotics, and implant retention) procedures. An extensive literature review was performed which identified 57 described cases of PJI. Transmission commonly occurred following scratches, bites and licks from cats and dogs. Patients often do not recognise the potential severity of these injuries and should be warned following arthroplasty of the risks of infection due to *Pasteurella* spp. following close contact with these animals, particularly given the increased morbidity and mortality associated with PJI.

## 1. Introduction

*Pasteurella* is a genus of Gram‐negative coccobacillus. They are small, facultatively anaerobic, nonmotile organisms that are primarily found as commensals in the mouths and on the skin of cats and dogs. Many members of this group are zoonotic pathogens, with *Pasteurella multocida* being the most commonly reported species causing human infection [1]. However, other species have also been reported to cause disease, including *Pasteurella canis*, *Pasteurella dagmatis*, *Pasteurella stomatis and Pasteurella aerogenes* [2]. Transmission commonly occurs as an outcome of close contact with animals, often resulting as a consequence of licks, bites and scratches from cats and dogs [3, 4]. Skin and soft tissue infection (SSTI) is the most predominant clinical manifestation of infection with *Pasteurella*, however, cases of pneumonia, meningitis, septic arthritis, and endocarditis due to *Pasteurella* are commonly reported, particularly among immunocompromised patients [5–8]. Although infections from *Pasteurella* species are infrequent, they have been reported as serious causes of bloodstream infections [2]. Infections in prosthetic joints make up a smaller proportion of infections but can be especially devastating, resulting in the need for surgical revisions and increased rates of mortality and morbidity [9].

## 2. Method

This retrospective study was conducted at two tertiary teaching hospitals within the same NHS trust with a total bed capacity of 1950 in Sheffield, United Kingdom. The microbiology laboratory electronic record system was searched for patients over 18 years old with positive *Pasteurella* spp. isolates from joint aspirates between 28 Feb 2013 and 28 Feb 2023. Medical records were reviewed for demographic and clinical data, including age, history, progression of illness, treatments and outcomes. *Pasteurella* species were identified using matrix‐assisted laser desorption/ionisation time‐of‐flight mass spectrometry (MALDI/TOF). We identified three cases of *Pasteurella* spp. isolated from joint aspirates in our centre. All three cases fit the European Bone and Joint Society (EBJIS) criteria definition of periprosthetic joint infection due to each case having more than 2 positive samples with the sample microorganism [10]. All patients described in these case reports have given their consent to be included.

## 3. Result

### 3.1. Case 1

A 61‐year‐old lady presented to A&E with a 1‐week history of left knee pain and yellow discharge from the surgical wound of a recent left total knee arthroplasty completed 5 weeks prior. She described episodes of minor trauma to the knee, such as hitting her knee against furniture and dropping a crutch on her knee, in the week prior to presentation. She was otherwise feeling generally well. She had a background of psychotic depression and factor XI deficiency. Her temperature was 37.6°C, BP 127/70, RR 20, HR 107 and SP02 was 96% in room air. On examination there was an area of erythema surrounding the surgical wound with serous blood‐stained fluid discharging from it. A diagnosis of septic arthritis was made, and she underwent a DAIR (debridement, antibiotics and implant retention) and knee aspiration procedure that day. During surgery it was noted that she had a markedly thickened synovium with a lot of fibrous tissue. She was started on IV aztreonam, metronidazole and teicoplanin on the advice of microbiology in the context of a penicillin allergy of a rash. Samples during surgery were taken for extended orthopaedic cultures. 3 days later samples were found to be positive for *P multocida*. On further discussion with the patient, she revealed that she had a pet dog that she regularly allowed to lick her wound. She continued to improve, and after 6 days of IV antibiotics, a multidisciplinary team (MDT) discussion decided she was to step down to oral ciprofloxacin 750 mg BD for 5 weeks and doxycycline 100 mg BD for 2 weeks. She was discharged on these antibiotics on day 7 of her admission. She was seen 1 month post‐discharge, where her wound was noted to be healing and she had increased range of movement of the knee. At this appointment she confirmed that she had asked her dog to lick her surgical wound to ease her symptoms after her initial procedure.

### 3.2. Case 2

An 81‐year‐old woman attended the accident and emergency (A&E) department with a 1‐week history of feeling generally unwell, confused and lethargic, resulting in a sudden collapse at home. She described erythema spreading from her ankle to knee over the course of two to 3 days. On admission, she was unable to move her knee. She was normally fit and independent with no significant past medical history except for bilateral total knee replacements 10 years prior to admission. On examination she had a swollen, tender right knee with well‐demarcated cellulitis in the whole lower limb. A small puncture wound on her lateral shin, oozing purulent pus, was also noted and, at the time, thought to be an insect bite. She was pyrexial with a temperature of 38.2 and tachycardic with an HR 107. Treatment for septic arthritis was initiated, and she started on IV piperacillin with tazobactam and teicoplanin before being taken to theatre for a washout of the joint and subsequent DAIR. During surgery, samples of turbid, foul‐smelling fluid were taken and sent to be cultured. Post‐operatively she required a 2‐day stay in HDU for vasopressor and inotropic support. During this time blood cultures were positive for *P. multocida*. Cultures from knee aspirates also grew *P. multocida* as well as coliform bacteria. After 2 days she was switched to IV co‐amoxiclav and then required a further 4 weeks of oral doxycycline. Her stay was prolonged and complicated by an upper GI bleed; however, she recovered well and was discharged back to her normal residence 1 month after initial admission. The patient was seen 1 month post‐discharge in an orthopaedic outpatient clinic. On examination her right knee was warm and swollen with reduced extension but no more than expected. The puncture wound on her right shin that was noted on initial presentation was found to be a result of a cat scratch that the patient did not think was significant at the time of presentation.

### 3.3. Case 3

A 47‐year‐old gentleman was admitted to the orthopaedic ward with right knee pain 1 month following a right total knee arthroplasty. He had a 3‐day history of increased right knee swelling and erythema with discharge from the surgical wound. He had a past medical history of gout, osteoarthritis, and previous Hartmann’s for diverticular abscess. He explained that the surgical wound had opened during a physiotherapy session 3 weeks prior and had been discharging fluid ever since. On examination the right knee was swollen, he had a dehiscent wound with discharge and was unable to flex or extend the knee. Observations taken were within normal range; however, bloods showed an increased CRP of 60. He was prepared for surgical knee aspiration and debridement and was operated on 2 days post‐admission. A superficial debridement was done with tissue samples sent for culture, and the team decided not to open the wound for further surgical exploration. Post‐operatively, he was started on IV cefuroxime and teicoplanin in the context of a penicillin allergy of a rash. Subjectively, the participant improved following surgery; however, the wound continued to discharge and remained swollen. Four days after the initial procedure, cultures of surgical samples were found positive for coagulase‐negative *Staphylococcus* and *P. canis*. He went back to surgery 7 days after the initial presentation and underwent aggressive debridement of the soft tissue and synovium with a washout. He received 2 weeks total of IV antibiotics and was discharged 1 week post‐surgery with 4 weeks of oral doxycycline 200 mg OD and flucloxacillin 500 mg QDS. Further discussions with the patient postoperatively revealed that he owned a dog that often slept in the same bed. It was determined that the infection most likely originated from cross‐contamination with the dog and secondarily from the dog licking the surgical wound. The patient’s wound healed appropriately within a normal time frame; however, he had problems with stiffness and pain for several years postoperatively. Three years after his initial TKR, he had a direct exchange of TKR, which resolved the long‐standing pain and stiffness.

## 4. Discussion

We report three cases of prosthetic joint infections (PJIs) due to *Pasteurella* spp., 2 of which resulted in DAIR procedures and 1 which was successfully treated with antibiotics and surgical lavage. In all three cases, a history of animal bite or scratch was noted and found to be the likely source of infection. Although uncommon, *Pasteurella* is an important zoonotic pathogen, with 600–700 laboratory‐confirmed cases reported in England and Wales each year [11]. *P. multocida* is of particular significance as it accounts for ~60% of infective cases. Transmission resulting in SSTI and joint infection primarily occurs from bites, scratches and licks from animals such as cats and dogs. Interestingly, animal exposure is less frequently identified in cases of invasive *Pasteurella* disease [12]. Compared with localised infection, invasive infections have an increased mortality rate and are more likely to occur in patients who have severe comorbidities and who are immunocompromised [13, 14]. In prosthetic joints, infection is a well‐known cause of clinical failure or postoperative complications. Some strains of *Pasteurella* are known to form a biofilm, increasing the likelihood of chronic or recurrent infection if the initial prosthetic infection is not adequately treated [15].

The table below demonstrates all case reports of *Pasteurella* infections in prosthetic joints found in our literature review (Table 1).

**Table 1:** All cases of *Pasteurella* PJI in the literature.

Study	Sex	Age	*Pasteurella* species	Affected joint	Comorbidities	Long term antibiotic suppression	Antibiotics used and duration	Nature of animal contact	Location of animal contact	Time from animal contact until symptom onset	Management	Long term outcomes
Griffin and Barber [16]	F	64	*P. multocida*	Knee	Rheumatoid arthritis	No	Ampicillin, duration not documented	Cat scratch	Shin (same side as joint)	3 days	Antibiotics, Nil surgery	Cure
Maurer et al. [17]	F	55	*P. multocida*	Knee	Rheumatoid arthritis	No	IV penicillin for 14 days	Dog lick	Unknown	Unknown	Antibiotics, Nil surgery	Cure
Sugarman et al. [18]	F	33	*P. multocida*	Knee	Rheumatoid arthritis	Yes‐ prolonged courses	1st episode: cloxacillin for “several months” 2nd episode:IV followed by oral penicillin for 14 months total	Dog lick	Leg (same side as joint)	Unknown	Antibiotics,prosthesis removal	Cure
Arvan and Goldberg [19]	F	72	*P. multocida*	Knee	None	Oral penicillin for 1 year	IV penicillin G for 3 weeks	Cat bite	Unknown	Unknown	Antibiotics, Surgical lavage and debridement, Prosthesis retained	Cure
Spagnuolo [20]	F	72	*P. multocida*	Knee	None	No	IV penicillin G for 3 weeks followed by oral penicillin for 4 weeks	Cat bite	Unknown	Unknown	Antibiotics, Surgical lavage and debridement, Prosthesis retained	Cure
Gomez‐Reino et al. [21]	F	64	*P. multocida*	Knee	Hypothyroidism	No	IV cephalothin for 6 weeks followed by oral cephalexin for undocumented period of time	Cat bite	Calf (same side as joint)	5 days	Antibiotics, Prosthesis removal	Cure
Mellors and Schoen [22]	F	62	*P. multocida*	Knee (bilateral)	None	Unable to access	Unable to access	Cat scratch	Unknown	Unknown	Antibiotics, Nil surgery	Cure
Orton and Fulcher [23]	F	74	*P. multocida*	Knee (bilateral)	None	No	IV ampicillin for 17days followed by oral penicillin and tetracycline for 3 months	Cat bite	Hand	1 day	Antibiotics, prosthesis removal	Cure
Gabuzda and Barnett [24]	F	88	*P. multocida*	Knee	None	Unable to access	Unable to access	Cat bite	Unknown	Unknown	Antibiotics, prosthesis removal and replacement	Cure
Guion and Sculco [25]	F	45	*P. multocida*	Knee	Rheumatoid arthritis	No	IV cefotaxime for 6 weeks	Dog scratch	Knee (same as joint)	1 week	Antibiotics, prosthesis removal and replacement	Cure
Braithwaite and Giddins [26]	F	48	*P. multocida*	Hip	Diabetes	No	IV penicillin and IV flucloxacillin for 4 weeks followed by oral penicillin and oral flucloxacillin for 4 weeks	Cat bite	Leg (same side as joint)	Unknown	Antibiotics, prosthesis removal and replacement	Cure
Maradona et al. [27]	F	73	*P. multocida*	Knee	Diabetes	No	IV pencillin G for 3 weeks followed by oral ciprofloxacin for 3 weeks	Dob bite	Calf (same side as joint)	65 days	Antibiotics, debridement, prosthesis retained	Cure
Antuña et al. [28]	F	73	*P. multocida*	Knee	Rheumatoid arthritis	No	IV ciprofloxacin for 6 weeks followed by IM ciprofloxacin for 4 weeks	Dog bite	Calf (same side as joint)	2 months	Antibiotics, surgical lavage and debridement, Prosthesis retained	Cure
Takwale et al. [29]	F	57	*P. multocida*	Hip	Rheumatoid arthritis	No	IV benzylpenicillin for 4 weeks followed by oral ciprofloxacin for 8 weeks	Cat scratch	Ankle (same side as joint)	2 weeks	Antibiotics, prosthesis removal and replacement	Cure
Chikwe et al. [30]	M	73	*P. multocida*	Hip	None	No	Not documented	Dog contact	Unknown	Unknown	Prosthesis removal and replacement	Unknown
Mehta and Mackie [31]	F	84	*P. multocida*	Hip	Rheumatoid arthritis	No	IV benzylpenicillin and oral ciprofloxacin for 1 week followed by oral ciprofloxacin for 7 weeks	Cat scratch	Ankle (same side as joint)	4 weeks	Antibiotics, prosthesis removal and replacement	Cure
Mehta and Mackie [31]	F	57	*P. multocida*	Hip	Rheumatoid arthritis	No	IV benzylpenicillin for 4 weeks followed by oral ciprofloxacin for 8 weeks	Cat scratch	Unknown	4 weeks	Antibiotics, prosthesis removal and replacement	Cure
Stiehl et al. [32]	M	63	*P. multocida*	Knee	None	No	Ciprofloxacin and piperacillin‐tazobactam, duration not documented	Dog contact and horse injury	Unknown	Unknown	Antibiotics, prosthesis removal and replacement	Cure
Polzhofer et al. [33]	F	73	*P. multocida*	Knee	None	No	IV ampicillin‐sulbactam and IV clindamycin for 3 weeks	Cat bite	Lower leg (same side as joint)	Unspecified days	Antibiotics, surgical lavage and debridement, prosthesis retained	Cure
Ciampolini et al. [34]	F	73	*P. multocida*	Knee	None	No	IV benzylpenicillin and oral ciprofloxacin for unclear length of time followed by oral amoxicillin and ciprofloxacin for 6 weeks	Cat scratch	Hand	2 weeks	Antibiotics, debridement, prosthesis removal and replacement	Cure
Zebeede et al. [35]	F	41	*P. multocida*	Knee	SLE and antiphospholipid syndrome	No	Ciprofloxacin for 3 months	Cat scratch	Second toe (same side as joint)	Unknown	Antibiotics, nil surgery	Cure
Heym et al. [36]	F	72	*P. multocida*	Knee	Prev appendectomy and prev hysterectomy	No	IV amoxicillin and oral doxycycline for 5 days followed by oral doxycycline and amoxicillin for 2 months	Dog lick	Toe (same side as joint)	Unspecified days	Antibiotics, prosthesis removal and replacement	Cure
Serrano et al. [37]	M	79	*P. multocida*	Knee	Dementia	No	IV amoxicillin‐clavulanic acid for 3weeks followed by ciprofloxacin for 4 weeks with co‐trimoxazole for 12 weeks	Cat scratch	Unknown	Unknown	Antibiotics, surgical lavage, prosthesis retained	Cure
Kadakia and Langkamer [38]	F	80	*P. multocida*	Knee	Breast carcinoma	No	IV cefuroxime for 2 weeks followed by 2 months of oral ciprofloxacin	Cat bite	Shin (same side)	6–8 days	Antibiotics, surgical lavage, prosthesis retained	Cure
Heydemann et al. [39]	M	66	*P. multocida*	Knee	Rheumatoid arthritis	No	Ceftriaxone for 4 weeks	Cat scratch	Unknown	7 days	Antibiotics, debridement, partial prosthesis removal and replacement	Cure
Mondon et al. [40]	F	77	*P. canis*	Knee	Ankylosing spondylitis	No	Levofloxacin and amoxicillin, duration not documented	Dog bite	Unknown	Unknown	Antibiotics, surgical lavage, prosthesis retained	Cure
Romanò et al. [41]	F	82	*P. multocida*	Knee	Rheumatoid arthritis	No	Amoxicillin‐clavulanic for 34 days and ciprofloxacin for 42 days	Cat scratch	Ankle and foot (same side)	5 months	Antibiotics, debridement, partial prosthesis removal and replacement	Cure
Miranda et al. [42]	M	64	*P. multocida*	Knee	HTN and hypercholesterolaemia	No	IV amoxicillin‐clavunate and levofloxacin for 10 days followed by oral amoxicillin‐clavunate and levofloxacin for 6 weeks	Cat bite and scratch	Lower leg (same side)	9 days	Antibiotics, surgical lavage, debridement, partial prosthesis removal and replacement	Cure
Subramanian et al. [43]	M	47	*P. multocida*	Knee	None	No	IV cefuroxime and teicoplanin (also treating S epidermis) for 2 weeks followed by oral doxycycline and clindamycin for 6 weeks	Dog contact	Knee (same as joint)	Unknown	Antibiotics, surgical lavage, debridement, partial prosthesis removal and replacement	Cure
Blanco et al. [44]	F	82	*P. multocida*	Knee	Rheumatoid arthritis	No	IV ciprofloxacin for 15 days followed by oral ciprofloxacin for 1 month	Cat scratch	Knee (same as joint)	4 days	Antibiotics, surgical lavage, debridement, prosthesis retained	Cure
Ferguson et al. [45]	F	67	*P. multocida*	Knee	None	No	Oral ciprofloxacin and IV amoxicillin for 2 weeks followed by 6 weeks of oral ciprofloxacin and amoxacillin	Dog lick	Legs (bilateral)	Unknown	Antibiotics, surgical lavage, debridement, partial prosthesis removal and replacement	Cure
Alsaffar and Guar [46]	F	74	*P. multocida*	Hip	Osteoporosis	No	Ciprofloxacin and IV amoxicillin for 6 weeks followed by 6 weeks of oral ciprofloxacin and amoxicillin	Cat bite	Lower leg (same side)	Unknown	Antibiotics, surgical lavage	Cure
Lam and Page [47]	F	55	*P. multocida*	Hip	Obesity	No	IV ertapenem and IV vancomycin (also treating corynebacterium striatum isolated) for 6 weeks	Dog lick	Lower leg (same side)	Unknown	Antibiotics, surgical lavage, debridement, prosthesis removal and replacement	Cure
Kouevidjin and Bassinga [48]	F	84	*P. multocida*	Knee	Diabetes, Obesity, HTN, prev PE, prev DVT	No	Oral Ofloxacin and amoxicillin for 2 months	Cat bite	Leg (same side as joint)	6 days	Antibiotics, surgical lavage, debridement, partial prosthesis removal and replacement	Cure
Costa‐Juan et al. [49]	F	83	*P. multocida*	Knee	HTN and hypercholesterolaemia	No	Levofloxacin IV for 2 weeks then orally for 6 weeks	Cat scratch	Ankle (unknown laterality)	4 days	Antibiotics, surgical lavage, prosthesis cleaned and retained	Cure
Ding et al. [50]	M	66	*P. multocida*	Shoulder	Common variable immunodeficiency syndrome, diabetes, prev lyme disease	No	Ceftriaxone for 6 weeks	Cat scratch	Unknown	Unknown	Antibiotics, surgical lavage, debridement, partial prosthesis removal and replacement	Cure
Schweon [51]	F	75	*P. multocida*	Hip	None	Not documented	Not documented	Cat contact	Unknown	Unknown	Antibiotics, prosthesis removal and replacement	Cure
Spelitz et al. [52]	M	82	*P. multocida*	Knee	CHD, Diabetes	No	Benzylpenicillin followed by oral ciprofloxacin and amoicillin/sulbactam, treatment length not documented	Cat bite	Calf (unknown laterality)	3 months	Antibiotics, Surgical lavage, debridement, prosthesis removal and replacement	Cure
Guilbart et al. [53]	M	74	*P. multocida*	Knee	AF, HTN, venous leg ulcers, obesity, alcoholism, aortic valve stenosis	No	Ampicillin and doxycycline followeed by piperacillin+tazobactam and vancomycin‐ciprofloxacin‐amikacin, treatment length not documented	Dog lick	Leg (same side as joint)	Unknown	Antibiotics (surgical prosthesis removal attempted)	Death
Honnorat et al. [54]	M	65	*P. multocida*	Knee	None	At least 6 months treatment given, exact length not documented	Amoxicillin and doxycycline, treatment length unknown	Dog lick	Unknown	Unknown	Antibiotics, surgical lavage and debridement, prosthesis retained	Cure
Honnorat et al. [54]	M	82	*P. multocida*	Hip	None	At least 6 months treatment given, exact length not documented	Amoxicillin and doxycycline, treatment length unknown	Cat scratch	Unknown	Unknown	Antibiotics, surgical lavage and debridement, prosthesis retained	Cure
Honnorat et al. [54]	F	63	*P. multocida*	Knee	Diabetes	At least 6 months treatment given, exact length not documented	Amoxicillin and doxycycline, treatment length unknown	Cat scratch	Unknown	Unknown	Antibiotics, surgical lavage, debridement, prosthesis removal and replacement	Cure
Honnorat et al. [54]	M	61	*P. multocida*	Knee	Diabetes and foot ulcers	At least 6 months treatment given, exact length not documented	Amoxicillin and doxycycline, treatment length unknown	Dog lick	Unknown	Unknown	Antibiotics, surgical lavage, debridement, prosthesis removal and replacement	Cure
Honnorat et al. [54]	F	81	*P. multocida*	Knee	Obesity	At least 6 months treatment given, exact length not documented	Amoxicillin and doxycycline, treatment length unknown	Cat scratch	Unknown	Unknown	Antibiotics, surgical lavage, debridement, prosthesis removal and replacement	Cure
Honnorat et al. [54]	F	85	*P. multocida*	Knee	None	At least 6 months treatment given, exact length not documented	Amoxicillin and doxycycline, treatment length unknown	Cat and dog contact	Unknown	Unknown	Antibiotics, surgical lavage and debridement, prosthesis retained	Cure
Arbefeville et al. [55]	M	80	*P. multocida*	Knee	AF, prev mitral and tricuspid valve repairs	No	Ceftriaxone for 6weeks	Dog contact	Unknown	Unknown	Antibiotics, prosthesis removal and replacement	Cure
Runnstrom et al. [56]	M	74	*P. multocida*	Knee	Obesity, AF, obstructive sleep apnoea, gout, non‐ischaemic cardiomyopathy with an implantable cardioverter‐defibrillator (ICD),	1 year oral pencillin V	Ampicillin‐sulbactam for unclear duration and then penicillin G continuous infusion for 6 weeks	Dog scratch	Unknown	Unspecified days	Antibiotics, surgical lavage, debridement, prosthesis removal and replacement	Cure
Fayyaz [57]	M	71	*P. multocida*	Hip	Alcohol abuse, COPD	No	Ampicillin‐sulbactam for 6 weeks	Non identified	n/a	n/a	Antibiotics, surgical lavage and debridement, prosthesis retained	Palliated
Lafont et al. [58]	F	92	*P. multocida*	Knee	None	No	IV ampicillin and oral levofloxacin for 12 days then levofloxacin to complete 12 weeks treatment	Cat scratch and lick	Unknown	Unknown	Antibiotics, surgical lavage, debridement, partial prosthesis removal and replacement	Cure
Abel et al. [59]	F	65	*P. multocida*	Shoulder	Multiple myeloma, prev autologous stem cell transplant	No	Ceftriaxone as inpatient then levofloxacin as outpatient for 6weeks total treatment	Cat lick	Wrist	Unknown	Antibiotics, surgical lavage, laminectomy, prosthesis retained	Cure
Smith and Sridhar [60]	F	63	*P. multocida*	Hip	HTN, Coronary artery disease, PVD, chronic back pain, depression	Doxycycline indefinitely	Ampicillin/sulbactam	Cat bite	Ankle (same side as joint)	Unspecified weeks	Antibiotics, surgical lavage, debridement, partial prosthesis removal and replacement	Post‐operative septic mycotic pseudoaneurysm secondary to contiguous spread of hip infection.
Denes et al. [61]	F	77	*P. multocida*	Hip	None	No	Ceftriaxone and ciprofloxacin for 6 weeks	Cat bite	Unknown	Unknown	Antibiotics, prosthesis removal and replacement	Cure
Shih and Chen [62]	M	52	*P. multocida*	Knee	Previous liver transplant	No	Ampicillin/sulbactam for 4 weeks	Cat scratch and bite	Ankle (same side as joint)	Unknown	Antibiotics, nil surgery	Cure
Maritati et al. [63]	F	82	*P. multocida*	Knee	AF, stroke, COPD, right mastectomy, left saphenectomy, bilateral vitrectomy	No	IV ceftriaxone, unclear length of time, then oral levofloxacin for 12 weeks	Dog lick	Ankles (bilateral)	Unknown	Antibiotics, surgical lavage and debridement, prosthesis retained	Cure
Ranavaya and Awadh [64]	F	75	*P. multocida*	Knee	Diabetes HTN	Oral co‐amoxiclav for 6 months	IV ceftriaxone for 6 weeks	Cat bite	Upper extremity	1 month	Antibiotics, prosthesis removal and replacement	Cure
Kuechly et al. [65]	M	70	*P. multocida*	Hip	Prev right femur fracture	No	IV ceftriaxone for 3 weeks. Antibiotics following two‐stage exchange	Cat contact	Unknown	Unknown	Antibiotics, prosthesis removal and replacement	Treatment failure, further debridement and two‐stage exchange required
Dombrowsky et al. [66]	F	74	*P. multocida*	Knee	HTN, hypothyroidism	No	IV ceftriaxone for 6 weeks	Cat scratch	Shin (same side as joint)	2 weeks	Antibiotics, surgical lavage, debridement, partial prosthesis removal and replacement	Cure

We conducted a search of the literature for all cases of prosthetic joint infection (PJI) due to *Pasteurella* and found in total 57 cases occurring between 1975 and 2024. 56 of these cases were due to infection by *P. multocida*, and in one case, the responsible organism was *P. canis* [40]. Therefore, to the best of our knowledge, the third case we present above is only the second ever case of PJI due to *P. canis*. No other species of *Pasteurella* has been identified in PJIs thus far, although species such as *P. dagmatis*, *P. stomatis and P. aerogenes* have been isolated in cases of bloodstream infections, endocarditis and SSTI [2, 67, 68]. The average age of patients in our review was 69.5 (± 12.1 years, range 33–92 years) and women were more commonly affected (38 of 57 cases). 42 out of the 57 cases occurred in prosthetic knees, making up the vast majority. However, other joints implicated included 13 prosthetic hip infections and 2 prosthetic shoulder infections.

In all but one case, some kind of animal contact was identified as the likely source of infection. Contiguous and haematogenous spread of the bacterium from an infectious wound to the affected prosthetic joint, predominantly due to cat and dog scratches or bites, were the most common modes of infection. We found in several cases that the location of the initial injury was at a site notably distant from the infected joint, for example, a scratch to the hand resulting in a prosthetic knee infection a few weeks later [34]. In one case however, infection was thought to be due to localised seeding of the prosthetic joint from latent infection from an old horse injury [32]. This highlights the ability of *Pasteurella* to cause both direct and haematogenous joint seeding in arthroplasty.

Timing from the moment of injury to the onset of symptoms varied greatly, from as little as 3 days up to 5 months. We frequently found that the likely cause of the infection was not discovered until after isolation of the bacterium and retrospective discussion with patients, where a more detailed history was taken. As with the second case presented above, patients often do not recognise the significance of injuries from animals or beloved pets and are less likely to associate animal injury with infection if the injury occurred a long time ago. In most cases patients presented with signs and symptoms in keeping with cellulitis and septic arthritis, such as joint swelling, erythema, and pain. Immunocompromise is a well‐established risk factor for all infections, including *Pasteurella* PJI, and the most common comorbidity was rheumatoid arthritis, where patients are often taking medications that act to suppress the immune system. A study by Denes et al. [61] investigated the immunology of a patient with chronic *P. multocida* PJI and found that she had a persistent T CD8+ lymphocytes deficit. They confirmed this same finding in 7 other cases of *P. multocida* SSTI, suggesting that having a persistent T CD8+ lymphocyte deficit may be an underlying contributing factor in infection with the bacterium [61].

Treatment of PJI due to *Pasteurella* commonly requires surgical intervention, including surgical lavage and debridement with adjunctive antibiotics, usually resulting in good outcomes and a cure. In two out of the three cases we present above, a DAIR procedure resulted in long‐term cure. In one case, the patient needed a further revision to resolve issues with chronic pain. We found in the literature that often a DAIR procedure was used as a first‐line treatment, and if this failed, the patient went on to have a curative total revision [36, 47, 65]. The decision to replace or retain the prosthesis is done on a case‐by‐case basis; however, in the only two cases that resulted in death or palliation, the prosthesis was retained [53, 57]. In both cases, the infection occurred in elderly comorbid patients, where less aggressive treatment options were sought initially, and the patient died before or during a planned attempt to remove the prosthesis. It is not clear if early removal of the prosthesis would have resulted in better outcomes in these cases. More research is needed to determine evidence‐based criteria for who should undergo a primary DAIR procedure vs prosthesis exchange following PJI.

In a few cases, patients were treated successfully with antibiotics and without any surgical intervention [16, 17, 22, 35, 61]. *Pasteurella* spp. infections are generally sensitive to multiple antibiotics, including penicillins, cephalosporins, and doxycycline [69, 70]. In 2 of the 3 cases described here, 2 antimicrobials were used for the prolonged oral antibiotic regime. Combination therapy of 2 antimicrobial agents is recommended in staphylococcal PJI, but there is no such recommendation in literature for gram‐negative causes of PJI. Fluoroquinolones are recommended for gram‐negative PJI associated with biofilm formation [71]. Current National Institute for Health and Care Excellence (NICE) guidelines recommend using co‐amoxiclav for 3 days as prophylaxis after an animal bite in patients with prosthetic joints [66]. In multiple cases, however, antibiotic prophylaxis at the time of animal encounter did not prevent a PJI from occurring, sometimes months after the initial injury. It is important, therefore, that clinicians are aware of the possibility of PJI due to zoonotic pathogens even if the initial bite or scratch was treated appropriately. One paper reported a case of PJI caused by a strain of *P. multocida* resistant to benzylpenicillin, ampicillin, and amoxicillin‐clavulanic acid [35]. Resistance among strains of *Pasteurella* may become a greater issue in the future as cases of *Pasteurella* PJI rise due to an expanding population of immunocompromised patients and an ageing population requiring more joint revisions.

## 5. Conclusion

*Pasteurella* is an important zoonotic pathogen causing PJI with potentially disastrous consequences. Patients with prosthetic joints, especially those who are immunocompromised, should be warned about the risks associated with close contact, licks, bites and scratches from animals such as cats and dogs. Those with animal injuries should receive prophylactic antibiotics to which *Pasteurella* spp. are susceptible. It is important that clinicians take a thorough history in cases of suspected PJI, which should include asking about any animal contact or injuries in the months preceding presentation. Each patient in our case reports had a good outcome and good recovery following diagnosis and treatment of their infection. It is our opinion that the best outcomes come from swift antibiotic and surgical treatment. However, further research is needed to assess outcomes from treatment of *Pasteurella* PJI with DAIR procedures vs joint revision.

## Conflicts of Interest

The authors declare no conflicts of interest.

## Funding

No funding was received for this research.

## Data Availability

Data sharing is not applicable to this article as no new data were created or analysed in this study.
